# Clinical features of autosomal recessive polycystic kidney disease in the Japanese population and analysis of splicing in *PKHD1* gene for determination of phenotypes

**DOI:** 10.1007/s10157-021-02135-3

**Published:** 2021-09-18

**Authors:** Shinya Ishiko, Naoya Morisada, Atsushi Kondo, Sadayuki Nagai, Yuya Aoto, Eri Okada, Rini Rossanti, Nana Sakakibara, China Nagano, Tomoko Horinouchi, Tomohiko Yamamura, Takeshi Ninchoji, Hiroshi Kaito, Riku Hamada, Yuko Shima, Koichi Nakanishi, Masafumi Matsuo, Kazumoto Iijima, Kandai Nozu

**Affiliations:** 1 Department of Pediatrics, Kobe University Graduate School of Medicine, 7-5-1, Kusunoki-cho, Chuo-ku, Kobe, Hyogo 650-0017 Japan; 2 Department of Clinical Genetics, Hyogo Prefectural Kobe Children’s Hospital, 1-6-7, Minatojimaminami-machi, Chou-ku, Kobe, Hyogo 650-0047 Japan; 3 Department of Nephrology, Hyogo Prefectural Kobe Children’s Hospital, 1-6-7, Minatojimaminami-machi, Chou-ku, Kobe, Hyogo 650-0047 Japan; 4 Department of Nephrology, Tokyo Metropolitan Children’s Medical Center, 2-8-29 Musashidai, Fichu-shi, Tokyo, 183-8561 Japan; 5 Department of Pediatrics, Wakayama Medical University, 811-1, Kimiidera, Wakayama, Wakayama 641-8509 Japan; 6 Department of Child Health and Welfare (Pediatrics), Graduate School of Medicine, University of Ryukyus, 207 Uehara, Nishihara-cho, Nakagami-gun, Okinawa, 903-2015 Japan; 7 KNC Department of Nucleic Acid Drug Discovery, Faculty of Rehabilitation, Kobe Gakuin University, 518 Arise Ikawadani-cho, Nishi-ku, Kobe Hyogo, 651-2113 Japan

**Keywords:** Autosomal recessive polycystic kidney disease, *PKHD1*, Hepatic fibrosis, Congenital hypothyroidism, Minigene assay

## Abstract

**Background:**

Autosomal recessive polycystic kidney disease (ARPKD) is caused by mutations in the *PKHD1* gene. The clinical spectrum is often more variable than previously considered. We aimed to analyze the clinical features of genetically diagnosed ARPKD in the Japanese population.

**Methods:**

We conducted a genetic analysis of patients with clinically diagnosed or suspected ARPKD in Japan. Moreover, we performed a minigene assay to elucidate the mechanisms that could affect phenotypes.

**Results:**

*PKHD1* pathogenic variants were identified in 32 patients (0–46 years). Approximately one-third of the patients showed prenatal anomalies, and five patients died within one year after birth. Other manifestations were detected as follows: chronic kidney disease stages 1–2 in 15/26 (57.7%), Caroli disease in 9/32 (28.1%), hepatic fibrosis in 7/32 (21.9%), systemic hypertension in 13/27 (48.1%), and congenital hypothyroidism in 3 patients. There have been reported that truncating mutations in both alleles led to severe phenotypes with perinatal demise. However, one patient without a missense mutation survived the neonatal period. In the minigene assay, c.2713C > T (p.Gln905Ter) and c.6808 + 1G > A expressed a transcript that skipped exon 25 (123 bp) and exon 41 (126 bp), resulting in an in-frame mutation, which might have contributed to the milder phenotype. Missense mutations in cases of neonatal demise did not show splicing abnormalities.

**Conclusion:**

Clinical manifestations ranged from cases of neonatal demise to those diagnosed in adulthood. The minigene assay results indicate the importance of functional analysis, and call into question the fundamental belief that at least one non-truncating mutation is necessary for perinatal survival.

**Supplementary Information:**

The online version contains supplementary material available at 10.1007/s10157-021-02135-3.

## Introduction

Autosomal recessive polycystic kidney disease (ARPKD) is an inherited cilia-related disorder characterized by the association of bilateral renal cystic disease and congenital hepatic fibrosis. The polycystic kidney and hepatic disease 1 (*PKHD1*) gene has been identified as the causative gene for ARPKD [1, 2], with 590 types of pathogenic mutation reported in The Human Gene Mutation Database to date (http://www.hgmd.cf.ac.uk, HGMD). *PKHD1*, extending over a genomic segment of at least 470 kb on chromosome 6p12, is one of the largest disease-causing genes in the human genome. The longest *PKHD1* transcript contains 67 exons that encodes a protein comprising 4074 amino acids. *PKHD1* encodes a single-transmembrane protein, called polyductin/fibrocystin, which is mainly expressed in the kidneys and liver. Fibrocystin localizes in the ciliary membrane, and it may be involved in regulating cell to cell adhesion and proliferation; it also acts as a membrane-bound receptor [1–3]. However, the detailed function of *PKHD1* and the onset mechanism of ARPKD are still unknown. Recently, mutations in *DAZ *interacting protein 1-like (*DZIP1L*) have been reported in patients with ARPKD, suggesting that ARPKD is not a homogeneous disorder and *DZIP1L* may also be involved in its pathogenesis [4], however, whether there is a causative relationship between *DZIP1L* and ARPKD requires further investigation [5].

The clinical spectrum of the disease is often more variable than previously considered [6–8]. Most cases have been identified either in utero or at birth. Approximately 30–50% of affected neonates die shortly after birth due to severe pulmonary hypoplasia and secondary respiratory insufficiency [9, 10]. In contrast, those who survive through the perinatal period express variable disease phenotypes, and some elderly patients with ARPKD are only moderately affected [8, 9]. A genotype–phenotype correlation has been reported in which patients with biallelic truncating mutations in *PKHD1* show a severe phenotype with perinatal demise, while children surviving the postnatal period carry at least one missense mutation [9]. However, some patients with missense mutations can present with a phenotype that is as severe as that associated with truncating mutations, suggesting that complex transcriptional alterations may play a role in defining the phenotype [11].

To date, no multi-center study has described the genotype and phenotype of genetically diagnosed ARPKD patients in Japanese populations. Therefore, we aimed to analyze the clinical features of patients who were referred to our institute for gene testing and genetically diagnosed with ARPKD. Additionally, we conducted a functional analysis using a minigene assay to reveal the existence of aberrant splicing caused by six mutations detected in our study to further investigate the genotype–phenotype correlation. One patient without a missense mutation survived the neonatal period, and this result was contradictory to previous reports. Thus, we aimed to elucidate the underlying mechanism that led to the milder phenotype despite the absence of a missense mutation. Additionally, three patients with one missense mutation died soon after birth. We aimed to evaluate whether these missense mutations affected splicing.

## Materials and methods

### Study design

We conducted gene testing using next-generation sequencing (NGS) in patients with clinically diagnosed or suspected ARPKD at Japanese hospitals between April 2016 and April 2021. We analyzed cases in which two or more *PKHD1* or *DZIP1L* variants were considered pathogenic. Detailed information regarding clinical features was obtained from the referring clinician or hospital records of patients.

### Genetic analysis

DNA was isolated from peripheral blood samples using a QuickGene Mini 80 system (Wako Pure Chemical Industries, Ltd., Tokyo, Japan) according to the manufacturer’s instructions. Direct sequencing or targeted sequencing using NGS was performed on the genes responsible for inherited renal diseases. For NGS, we used a HaloPlex HS or SureSelect (Agilent Technologies, Santa Clara, CA, USA) according to the manufacturer’s instructions, and sequencing was performed using the MiSeq platform (Illumina, San Diego, CA, USA). HaloPlex HS was used for targeted sequencing of 128 (version 2, Supplementary Table 1), 172 (version 4, Supplementary Table 2), 159 (version 5, Supplementary Table 3), 164 (version 6, Supplementary Table 4), and 181 (version 7, Supplementary Table 5) genes, and SureSelect was used for the targeted sequencing of 203 (version 8, Supplementary Table 6) and 193 genes (version 9, Supplementary Table 7) associated with congenital anomalies of the kidney and urinary tract, and various cystic kidney diseases, including ARPKD, autosomal dominant polycystic kidney disease, and nephronophthisis, as cataloged in OMIM (https://www.omim.org) or PubMed (https://pubmed.ncbi.nlm.nih.gov) database.

Data were analyzed using SureCall 4.0 (Agilent Technologies), a software for end-to-end NGS data analysis. The cDNA reference numbers of *PKHD1* and *DZIP1L* were NM_138694.3 and 173,543.2, respectively. Pathogenicity predictions were performed in accordance with the guidelines of the American College of Medical Genetics (Supplementary Table 8). Several websites, including CADD (https://cadd.gs.wa shington.edu/), PROVEAN (http://provean.jcvi.org/index. php), SIFT (https://sift.bii.a-star.edu.sg/), PolyPhen-2 (http://genetics.bwh.harvard.edu/pph2/), and Mutation Taster (http://www.pathogenic varianttaster.org/) were used to predict variant pathogenicity (Supplementary Table 9). The splice sites of each variant were predicted using Human Splicing Finder (https://hsf.genomnis.com/home). Pair analysis using SureCall was used to determine the changes in copy number relative to a reference [12]. Changes in copy number were confirmed by multiplex ligation and probe amplification (MLPA) using SALSA P341-B4/P342-C1 PKHD1 (MRC-Holland, Amsterdam, the Netherlands), as suggested by the manufacturer. The MLPA test was performed twice to confirm abnormal changes.

### Minigene assay

We conducted in vitro analysis using a minigene assay for the following: missense mutations, c.9533G > T (p.Gly3178Val) in SC293, c.3944 T > G (p.Leu1315Arg) in SC324, and c.983G > A (p.Arg328Gln) in SC589; splice site mutations, c.8555-2A > C in SC324 and c.6808 + 1G > A in SC499; and nonsense mutation, c.2713C > T(p.Gln905Ter) in SC499. To create hybrid minigene constructs, we used the previously developed H492 vector, which is based on the pcDNA 3.0 mammalian expression vector (Invitrogen, Carlsbad, CA, USA) [13]. We cloned DNA fragments from both wild-type and patient peripheral leukocytes containing exons and introns around the target variants in *PKHD1* gene using In-Fusion cloning methods with the HD Cloning Kit (Takara Bio Inc., Kusatsu, Japan) according to manufacturer’s instructions (Supplementary Fig. 1). Primers used for cloning in the minigene assay for each mutation are listed in Supplementary Table 10. The hybrid minigenes were confirmed by sequencing, and they were transfected into HEK293T cells using Lipofectamine^®^ 2000 (Thermo Fisher Scientific, Waltham, MA, USA). Total RNA was extracted from cells after 24 h using the RNeasy Plus Mini Kit (QIAGEN, Hilden, Germany). Total RNA was reverse-transcribed using ReverTra Ace (Toyobo, Osaka, Japan). PCR was performed using a forward primer corresponding to a segment upstream of exon A and reverse primer complementary to a segment downstream of exon B, as previously described. PCR products were analyzed via electrophoresis on a 1.5% agarose gel using a DNA ladder, and this was followed by direct sequencing.

## Results

### Patient characteristics

*PKHD1* pathogenic variants were identified in 32 patients from 31 families. The *DZIP1L* mutation was not detected in any patient. There were 9 men and 23 women, and the median age of patients at the time of gene testing was 5 years (0–46 years) (Table 1). The patients were recruited from 26 hospitals in Japan.

**Table 1:** Patient characteristics

	Patients (*n* = 32)	T/T	T/NT	NT/NT
Age at suspected diagnosis				
Median	4 months			
Range	GA 25w – 36 years			
Age at genetic diagnosis				
Median	5 years			
Range	0 day–46 years			
Gender				
Male	9	1	5	3
Female	23	2	15	6
Kidney function*				
CKD stage 1	9/26 (34.6%)		5	4
CKD stage 2	6/26 (23.1%)		4	2
CKD stage 3	5/26 (19.2%)		5	
CKD stage 4	2/26 (7.7%)		2	
CKD stage 5	0/26 (0%)			
Renal replacement therapy	4/26 (15.44%)	2		2
Hepatic disease^a^				
Caroli disease	9/32 (28.1%)	2	5	2
Hepatic fibrosis	7/32 (21.9%)		3	4
Hepatic cysts	2/32 (6.2%)		1	1
Other manifestations^a^				
Hypertension (children)	12/23	2	7	3
Hypertension (adult)	1/4		1	
Respiratory failure at birth	7	2	4	1
Urinary tract infection	4		4	
Congenital hypothyroidism	3		1	2
Urolithiasis	2			2
Thrombocytopenia	1			1
Splenomegaly	1			1
Vesicoureteral reflux	1		1	
Perthes disease, inguinal hernia	1			1

*CKD* chronic kidney disease, *GA* gestational age, *NT* non-truncating mutation, *T* truncating mutation, *w* weeks

^a^Only the number of evaluable patients is shown

### PKHD1 mutations

In total, 64 mutations were identified. SC481 and her elder brother harbored the same mutations, and two patients (SC746 and SC756) had homozygous mutations; thus, we analyzed 60 variants, of which, 58 were detected via NGS and confirmed via direct sequencing, and two were detected via MLPA. Among the 60 variants, 34 missense mutations (56.7%), 17 nonsense mutations (28.3%), four disruption of a conserved splice site (6.7%), three frameshift mutations (5.0%), and two large deletions (3.3%) were detected. Among the point mutations, 20 variants were novel mutations in HGMD, dbSNP, and ClinVar (Table 2). c.5174G > C (p.Trp1725Ser), c.6794A > T (p.His2265Leu), c.7867delT (p.Try2623Thrfs*44), and c.9533G > T (p.Gly3178Val) genes were detected in multiple patients (Table 2, Supplementary Table 9).

**Table 2:** Genotypes and clinical manifestations of patients

Family	Patient	Age at gene testing	Gender	genotype	Exon	Amino acid	Inheritance	Mutation	HGMD	Clin Var	dbSNP	NGS panel
1	SC272	5	M	c.2507 T > C	24	p.Val836Ala	Unknown	Missense	CM144037	–	rs199568593	2
				c.9008C > T	58	p.Ser3003Phe	Maternal	Missense	CM1511302	–	–	
2	SC282	5	F	c.11G > A	2	p.Trp4Ter	Unknown	Nonsense	–	RCV000673461.3	–	2
				c.2507 T > C	24	p.Val836Ala	Unknown	Missense	CM144037	RCV000788709.1	rs199568593	
3	SC293	0	F	c.7113 T > G	45	p.Tyr2371Ter	Paternal	Nonsense	–	–	–	2
				c.9533G > T	58	p.Gly3178Val	Maternal	Missense	–	–	–	
4	SC324	0	F	c.3944 T > G	32	p.Leu1315Arg	Maternal	Missense	-	–	–	4
				c.8555-2A > C	IVS 54		Paternal	Splice site	-	RCV000493982.1	–	
5	SC331	8	F	c.7396G > T	47	p.Glu2466Ter	Unknown	Nonsense	–	–	–	4
				c.8859G > C	57	p.Leu2953Phe	Unknown	Missense	–	–	–	
6	SC365	2	M	c.274C > T	4	p.Arg92Trp	Unknown	Missense	CM100442	RCV000337196.1	rs370277502	4
				c.9319C > T	58	p.Arg3107Ter	Unknown	Nonsense	CM032330	RCV000169496.7	rs786204688	
7	SC410	7 m	F	c.3467C > T	30	p.Ser1156Leu	Maternal	Missense	CM051143	RCV001027935.1	rs367707903	4
				c.5585C > A	34	p.Ser1862Ter	Paternal	Nonsense	–	–	–	
8	SC432	2 w	F	c.1486C > T	16	p.Arg496Ter	Maternal	Nonsense	CM032309	RCV000004330.9	rs137852949	4
				c.6840G > A	42	p.Trp2280Ter	Paternal	Nonsense	CM1620515	RCV001209271.1	–	
9	SC443	46	F	c.2507 T > C	24	p.Val836Ala	Maternal	Missense	CM144037	RCV000779513.5	rs199568593	5
				c.8566A > T	55	p.Lys2856Ter	Unknown	Nonsense	–	–	–	
10	SC481	27	F	c.11611 T > C	65	p.Trp3871Arg	Maternal	Missense	CM051193	RCV001004185.1	rs754626014	5
				c.11881C > T	67	p.Arg3961Ter	Paternal	Nonsense	CM1925852	–	rs144193508	
	Brother	30	M	c.11611 T > C	65	p.Trp3871Arg	Maternal	Missense	CM051193	RCV001004185.1	rs754626014	
				c.11881C > T	67	p.Arg3961Ter	Paternal	Nonsense	CM1925852	RCV000665966.4	rs144193508	
11	SC488	1 m	F	c.977-3C > G	IVS 13		Unknown	Splice site	–	–	–	5
				c.10180 T > C	61	p.Cys3394Arg	Unknown	Missense	CM1612128	–	–	
12	SC494	5	F	c.5174G > C	32	p.Trp1725Ser	Unknown	Missense	–	–	rs761046498	6
				(*PKHD1* exons54-55) × 1			Unknown	Large deletion	–	–	–	
13	SC498	16	F	c.5174G > C	32	p.Trp1725Ser	Unknown	Missense	–	–	rs761046498	6
				c.7867delT	49	p.Tyr2623Thrfs*44	Unknown	Frameshift	–	–	–	
14	SC499	4	F	c.2713C > T	25	p.Gln905Ter	Unknown	Nonsense	CM1514390	RCV001243159.1	–	6
				c.6808 + 1G > A	IVS 41		Unknown	Splice site	–	–	–	
15	SC528	4 m	F	c.7237C > T	46	p.Arg2413Cys	Maternal	Missense	–	–	rs553534988	6
				c.8893 T > C	57	p.Cys2965Arg	Paternal	Missense	CM054807	RCV000672675.1	rs770068023	
16	SC529	11 m	F	c.1836 + 1G > A	IVS 19		Maternal	Splice site	–	RCV001004210.2	rs780898021	6
				c.5935G > A	37	p.Gly1979Arg	Paternal	Missense	CM127371	–	–	
17	SC567	6	M	c.5174G > C	32	p.Trp1725Ser	Paternal	Missense	–	–	rs761046498	7
				c.11456delT	64	p.Leu3819Ter	Maternal	Nonsense	–	–	–	
18	SC574	6 m	F	c.4292G > A	32	p.Cys1431Tyr	Unknown	Missense	CM149116	RCV000675159.4	rs753307105	7
				c.9533G > T	58	p.Gly3178Val	Unknown	Missense	–	–	–	
19	SC583	5 m	F	c.865C > T	12	p.Gln289Ter	Maternal	Nonsense	–	–	–	7
				c.5935G > A	37	p.Gly1979Arg	Paternal	Missense	CM127371	–	–	
20	SC589	0	F	c.983G > A	14	p.Arg328Gln	Paternal	Missense	CM149111	RCV000734720.1	rs770494581	7
				c.8011C > T	50	p.Arg2671Ter	Maternal	Nonsense	CM020499	RCV000004328.5	rs137852947	
21	SC601	6	F	c.1421A > C	16	p.His474Pro	Unknown	Missense	–	–	–	7
				c.5174G > C	32	p.Trp1725Ser	Unknown	Missense	–	–	rs761046498	
22	SC619	13	M	c.2725C > T	26	p.Arg909Ter	Unknown	Nonsense	CM1920176	RCV000176696.6	–	8
				c.5935G > A	37	p.Gly1979Arg	Unknown	Missense	CM127371	–	–	
23	SC637	13	M	c.11G > A	2	p.Trp4Ter	Paternal	Nonsense	–	RCV000673461.3	–	8
				c.6794A > T	41	p.His2265Leu	Maternal	Missense	–	–	–	
24	SC681	18	F	c.2507 T > C	24	p.Val836Ala	Unknown	Missense	CM144037	RCV000788709.1	rs199568593	8
				c.10414 T > G	61	p.Cys3472Gly	Maternal	Missense	–	RCV001052108.1	–	
25	SC697	0 m	M	c.7867delT	49	p.Tyr2623Thrfs*44	Paternal	Frameshift	–	–	–	8
				(*PKHD*1 exon50) × 1			Maternal	Large deletion	–	–	–	
26	SC704	23	F	c.5935G > A	37	p.Gly1979Arg	Unknown	Missense	CM127371	–	–	8
				c.7867delT	49	p.Try2623Thrfs*44	Unknown	Frameshift	–	–	–	
27	SC746	4 m	F	c.9764G > C	58	p.Trp3255Ser (homozygous)	Unknown	Missense	–	–	–	8
28	SC756	9	M	c.9107 T > G	58	p.Val3036Gly (homozygous)	Parental	Missense	CM034281	RCV000729595.1	rs893497345	9
29	SC772	11	F	c.1396G > A	16	p.Gly466Arg	Unknown (not maternal)	Missense	CM188344	–	rs1410954062	9
				c.6794A > T	41	p.His2265Leu	Maternal	Missense	–	–	rs1554300376	
30	SC791	16	M	c.2507 T > C	24	p.Val836Ala	Unknown	Missense	CM144037	RCV000788709.1 etc	rs199568593	9
				c.5780G > A	36	p.Arg1927Lys	Unknown	Missense	–	–	rs1485642148	
31	SC793	5 m	F	c.1690C > T	18	p.Arg564Ter	Maternal	Nonsense	CM100548	RCV001174805.2	rs765251347	9
				c.2507 T > C	24	p.Val836Ala	Paternal	Missense	CM144037	RCV000788709.1 etc	rs199568593	

Family	Patient	Age at gene testing	eGFR (RRT)	Hepatic disease	Hypertension	Other manifestations	Age when suspected	First consultation	Manifestations observed at first consultation	Prognosis
1	SC272	5	N/A	Caroli disease Hepatic fibrosis	N/A	Thrombocytopenia, splenomegaly	1	Medical consultation	Abdominal distention, hepatomegaly	Alive
2	SC282	5	116	Caroli disease	–	–	3 m	Medical check-up for infants	Kidney enlargement	Alive
3	SC293	0	N/A	N/A	N/A	Respiratory failure	GA 28 w	Fetal US	Oligohydramnios, high echogenicity in kidney	died at day 2
4	SC324	0	N/A	N/A	N/A	Respiratory failure	Perinatal	Fetal US	Oligohydramnios	died at day 1
5	SC331	8	106.6	Hepatic fibrosis, Caroli disease	–	Right vesicoureteral reflex	7	Urinary tract infection	Kidney enlargement, intrahepatic bile duct dilatation	Alive
6	SC365	2	92.3	Hepatic fibrosis	+	Urinary tract infection	5 m	RS virus infection	Hypertension	Alive
7	SC410	7 m	94.9	–	+	–	7 m	Vomiting, Diarrhea	Kidney enlargement	Alive
8	SC432	0 m	sCr 2.65	–	+	–	GA 25 w	Fetal US	Oligohydramnios, kidney enlargement, cystic kidney	Alive
9	SC443	46	24.4	Caroli disease	–	–	36	Medical check-up at workplace	Hyper echogenicity in kidney, intrahepatic bile duct dilatation	Alive
10	SC481	27	16.7	–	–	–	23	Medical check-up at workplace	Kidney dysfunction	Alive
	Brother	30	35.3	–	–	–	24	Medical check-up at workplace	Kidney dysfunction	Alive
11	SC488	1 m	NA	–	+	Hypothyroidism	Perinatal	Fetal US	Oligohydramnios, intrauterine growth restriction	Alive
12	SC494	5	79.7	Caroli disease	+	Urinary tract infection	10 m	Medical consultation for roseola and cystitis	Kidney enlargement, polycystic kidney	Alive
13	SC498	16	34.7	–	–	–	16	School urinalysis	Polycystic kidney, kidney dysfunction	Alive
14	SC499	4	N/A (PD 6 m-)	Caroli disease	–	Severe developmental delay due to neonatal asphyxia	Soon after birth	Respiratory failure	Kidney enlargement	Alive
15	SC528	4 m	125.1	–	–	Hypothyroidism, hyponatremia	Perinatal	Fetal US	Oligohydramnios	Alive
16	SC529	11 m	96	–	–	–	4 m	Medical check-up for infants	Kidney enlargement	Alive
17	SC567	6	35.2	–	+	Acute myeloid leukemia (4 y), Kawasaki disease (6 y), hyperuricemia	Perinatal	Fetal US	Cystic kidney	Alive
18	SC574	6 m	N/A(CHD 0d−4 m, PD 3w−)	Caroli disease	+	Hypothyroidism, hypocarnitinemia, hypozincemia	GA 33 w	Fetal US	Oligohydramnios, kidney enlargement	Alive
19	SC583	5 m	66	–	+	Congestive cardiac failure	4 m	Routine vaccination	Abdominal distension hypertension	Alive
20	SC589	0	NA	N/A	N/A	Respiratory failure	GA 29 w	Fetal US	Oligohydramnios, kidney enlargement, cystic kidney	died at day 0
21	SC601	6	76.3	–	+	–	5	Medical consultation	Hypertension	Alive
22	SC619	13	37.7	Caroli disease, recurrent cholangitis	+	Right Perthes disease, bilateral inguinal hernia	Soon after birth	Respiratory failure	Kidney enlargement, cystic kidney	Alive
23	SC637	13	71.5	–	–	–	11	Urinary tract infection	Kidney enlargement, polycystic kidney	Alive
24	SC681	18	107.4	Hepatic fibrosis	–	–	18	Medical check-up at school	Cystic kidney, hepatic fibrosis	Alive
25	SC697	0 m	sCr 2.4(HD 0 m, PD 0–2 m)	Caroli disease, acute cholangitis	+	Respiratory failure	GA 26w	Fetal US	Oligohydramnios, kidney enlargement, hyper echogenicity in kidney	Died at 2 m
26	SC704	23	30.3	Hepatic fibrosis	+	Autism, ADHD, mild intellectual disability	22	Annual visit for psychiatrist	Kidney dysfunction	Alive
27	SC746	4 m	(HD 0 m)	Hepatic fibrosis	+	Respiratory failure	GA 32 w	Fetal US	Oligohydramnios, kidney enlargement, hyper echogenicity in kidney	died at 5 m
28	SC756	9	171.2	Hepatic fibrosis	N/A	–	unknown	unknown	unknown	Alive
29	SC772	11	75.6	–	–	-	11	School urinalysis	Cystic kidney kidney enlargement, urolithiasis	Alive
30	SC791	16	110.4	Hepatic cysts	–	–	16	Lateral abdominal pain	Cystic kidney, Cystic liver, microscopic hematuria urolithiasis	Alive
31	SC793	5 m	78.3	Hepatic cysts	–	–	3 m	Enlarged kidney	Cystic kidney, cystic liver	Alive

*ACMG* American College of Medical Genetics, *ADHD* attention deficit hyperactivity disorder, *CHD* continuous hemodialysis, *d* day, *eGFR* estimated glomerular filtration rate, *F* female, *GA* gestational age, *HD* hemodialysis, *M* male, *m* month, *N/A* not available, *NGS* next-generation sequencing, *PD* peritoneal dialysis, *PM* moderate evidence of pathogenicity, *PP* supporting evidence of pathogenicity, *PS* strong evidence of pathogenicity, *PVS* very strong evidence of pathogenicity, *RRT* renal replacement therapy, *sCr* serum creatinine, *US* ultrasonography, *w* weeks, *y* years

### Clinical features

Ten of the 32 patients (31.3%) showed prenatal anomalies with oligohydramnios (*n* = 9/10), kidney enlargement (*n* = 5/10), cystic kidney (*n* = 3/10), or increased renal echogenicity (*n* = 3/10). Three of these patients (SC293, SC324, and SC589) died soon after birth due to respiratory failure, and two patients (SC697 and SC746) died within the first year of life. Two patients were suspected to have ARPKD soon after birth following the detection of respiratory failure and enlarged kidneys. The remaining patients were primarily diagnosed or suspected incidentally after the neonatal period, especially through medical checkups for infants or at school (*n* = 3), or workplace (*n* = 3) and school urinary screening (*n* = 2). Another reason for the initial visit to doctors was urinary tract infection (*n* = 2). The most common manifestation leading to diagnosis in pediatric cases was enlarged kidneys while in all the four adult cases, kidney dysfunction led to a diagnosis. The occasions for consultation and manifestations are presented in Table 2.

**Fig. 1:**
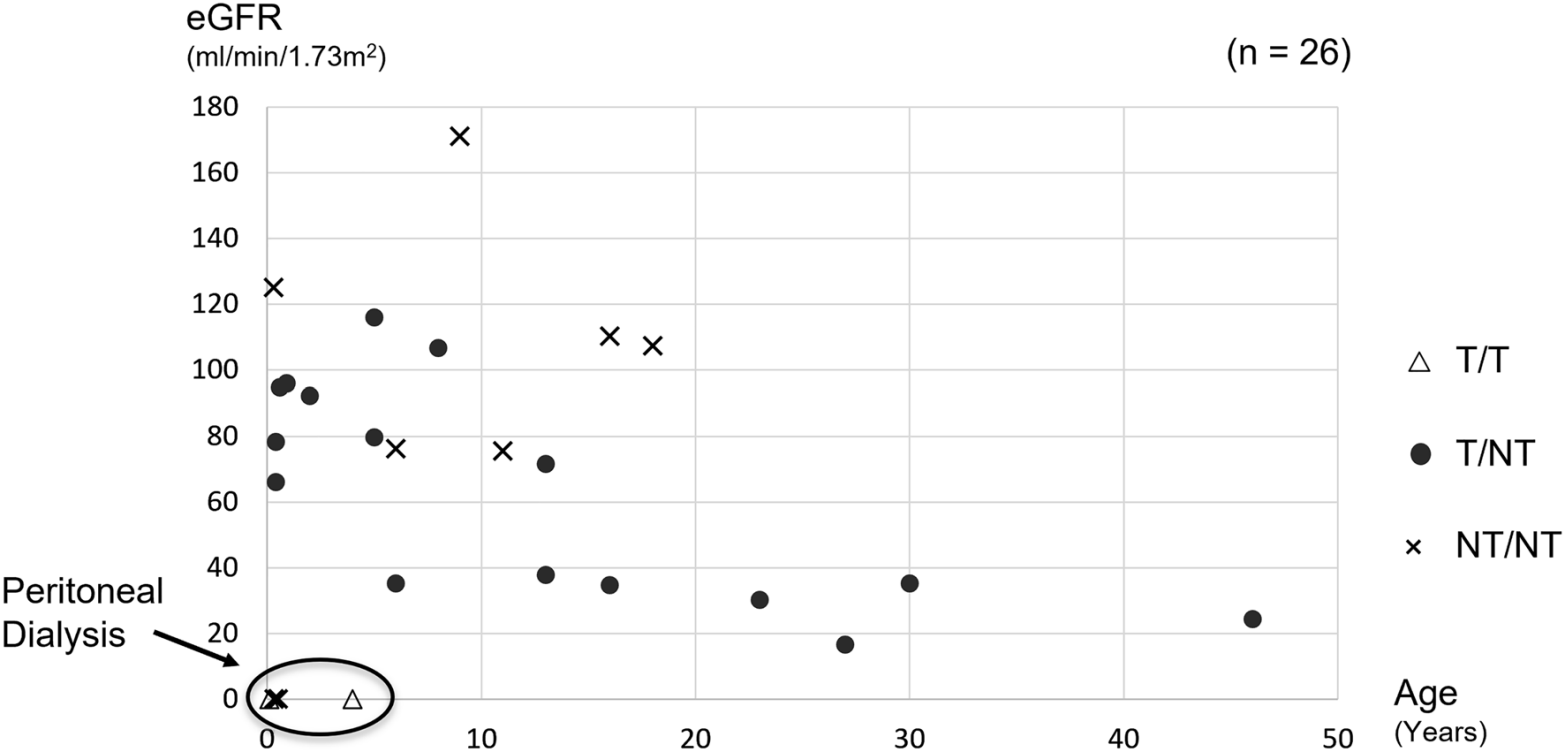
The table shows eGFR of each case with the available data. Kidney functions varied among pediatric patients, and four patients underwent peritoneal dialysis while adult patients showed severe kidney dysfunction. Two patients with truncating mutations in both alleles underwent peritoneal dialysis. Six of eight patients with two missense mutations were at CKD stage 1 or 2, but two of them needed renal replacement therapy from a young age. *T* truncating mutation, *N/T* non-truncating mutation, *CKD* chronic kidney disease, *eGFR* estimated glomerular filtration rate

More than half of the patients (*n* = 15/26) at the analyzed visit showed native kidney function in chronic kidney disease (CKD) stage 1 or 2. Four patients underwent peritoneal dialysis, and three of them were required to undergo hemodialysis during the neonatal period. None of the patients underwent kidney transplantation at the time of genetic analysis. The distribution between patients’ age and kidney functions, classified by the mutation type, is shown in Fig. 1.

Among the 27 surviving cases, imaging findings via ultrasonography and/or CT scan detected Caroli disease in 8 of 27 patients (29.6%) and hepatic fibrosis in 6 patients (22.2%). One patient underwent liver transplantation at the age of 14 years (SC619). Thrombocytopenia and splenomegaly due to portal hypertension were detected in one patient (SC272). Almost half of the pediatric patients (*n* = 12/23) showed systemic hypertension while one of the four adult patients did. Congenital hypothyroidism was observed in three patients in the neonatal mass-screening test, and treatment with levothyroxine was required.

### Genotype–phenotype correlation in *PKHD1*

Nine patients harbored missense mutations on both alleles, and all of them survived the neonatal period, although one patient (SC746) died at the age of 5 months. Additionally, 21 patients had one missense mutation, and three of them died during the neonatal period due to respiratory failure (SC293, SC324, and SC589). Three patients had no missense mutations (SC432, SC499, and SC697), and all patients were diagnosed at an early stage in their life (Fig. 1).

## Functional analysis via minigene assay

**Fig. 2:**
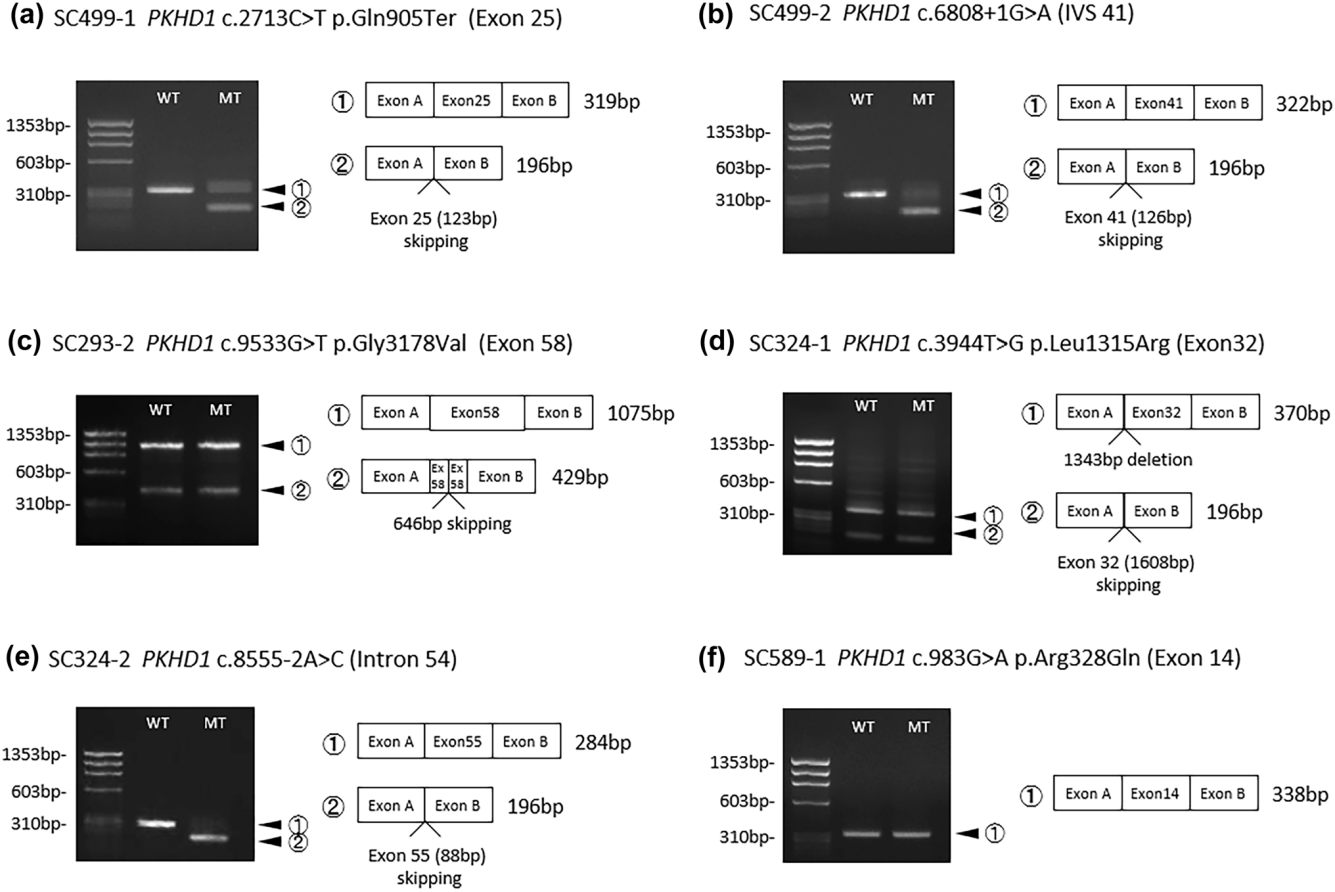
Reverse transcription-polymerase chain reaction amplified products of minigene transcripts. **a** c.2713C > T (SC499-1) minigene expressed a full-length transcript in WT and a transcript that skipped exon 25 in MT. **b** c.6808 + 1G > A (SC499-2) minigene expressed a full-length transcript in WT, and a transcript that skipped exon 41 in MT. **c** c.9533G > T (SC293-2) minigene mainly expressed a full-length transcript, and a few transcripts exhibiting 646 bp deletion in exon 58 in both WT and MT. **d** c.3944 T > G (SC324-1) minigene mainly expressed a transcript exhibiting 1343 bp deletion in exon 32, exon 32 skipping, and multiple thin bands that could not be sequenced in both WT and MT. **e** c.8555-2A > C (SC324-2) minigene expressed a full-length transcript in WT, and a transcript exhibiting exon 55 skipping in MT. f) c.983G > A (SC589-2) minigene expressed a full-length transcript in both WT and MT. WT, wild type. *MT* mutant

Assays using the minigene system were performed using gDNA fragments from four patients (SC293, SC324, SC499, and SC589). The electrophoresis results of minigene transcripts for each mutation are shown in Fig. 2. Additionally, the in silico analysis and clinical course of each patient are shown in Table 3. The inserted sequences for each case are shown in Supplementary Fig. 2, and direct sequencing of the minigene transcript of each mutation is shown in Supplementary Fig. 3. For both mutations in SC499, which had no missense mutation (c.2713C > T, c.6808 + 1G > A), both minigenes expressed a transcript that skipped an exon, in which the number of base pairs was a multiple of three, accompanied by multiple transcripts similar to the wild type transcript size, in smaller amounts. On the other hand, for SC293, SC324, and SC589, with one missense mutation associated with perinatal demise, every minigene expressed a normal transcript similar to the wild type. Additionally, for the splice site mutation (c.8555-2A > C) in SC 324, each minigene expressed a transcript that skipped exon 55.

**Table 3:** Results of minigene assay and in silico analysis, and clinical course of patients with mutations conducted for minigene assay

Case	cDNA	Amino acid	Exons	Mutation	Results of minigene assay	In silico analysis by human splicing Finder	Clinical course
SC499-1	c.2713C > T	p.Gln905Ter	25	Nonsense	Exon 25 skipping (123 bp)	No significant impact on splicing signals	Incubation after birth. Extubation at 5 months. Right nephrectomy at 6 months, and following this, peritoneal dialysis was initiated. Left nephrectomy at 1 year and 4 months
SC499-2	c.6808 + 1G > A	−	IVS 41	Splice site	Exon 41 skipping (126 bp)	Alteration of the WT donor site, most probably affecting splicing	
SC293-1	c.7113 T > G	p.Tyr2371Ter	45	Nonsense	−	Significant alteration of ESE/ESS motifs ratio Activation of a cryptic acceptor site. Potential alteration of splicing	Died due to respiratory failure at day 2
SC293-2	c.9533G > T	p.Gly3178Val	58	Missense	Same transcript as wild type	Significant alteration of ESE/ESS motifs ratio. Activation of a cryptic acceptor site. Potential alteration of splicing	
SC324-1	c.3944 T > G	p.Leu1315Arg	32	Missense	Same transcript as wild type	No significant impact on splicing signals	Died due to respiratory failure at day 2
SC324-2	c.8555-2A > C	-	IVS 54	Splice site	Exon 55 skipping (88 bp)	Alteration of the WT acceptor site, most probably affecting splicing	
SC589-1	c.983G > A	p.Arg328Gln	14	Missense	Same transcript as wild type	No significant impact on splicing signals	Died due to respiratory failure at day 0
SC589-2	c.8011C > T	p.Arg2671Ter	50	Nonsense	–	Significant alteration of ESE/ESS motifs ratio	

*ESE* exonic splicing enhancer, *ESS* exonic splicing silencer, *WT* wild type

## Discussion

This is the first multicenter report of genetically diagnosed ARPKD in the Japanese population. Although large-scale studies on ARPKD have been conducted in Europe and North America [7–10, 14], no study has been reported from Japan. In this study, 6 cases (19.4%) were suspected to have ARPKD prenatally while the others were suspected or diagnosed after birth in various situations, mostly incidentally, which is in accordance with previous studies reporting that postnatal accidental finding is the most common reason for the initial visit [7, 8]. Screening urinalysis at school is performed every year in Japan, and this system enabled the diagnosis of ARPKD in two patients (SC498 and SC772). Almost half of the patients at the analyzed visit showed kidney functions within CKD stages 1 or 2 while four patients underwent renal replacement therapy. This finding suggests that the surviving patients show various phenotypes in the kidney, as previously described [9]. With the improvement of prognosis and advancement of renal insufficiency management, hepatobiliary disease is likely to become more prevalent. Caroli disease was observed in almost one-third of the patients, which is in accordance with previous reports [8, 10, 15]. In two cases of cholangitis, one patient underwent liver transplantation because of recurrent episodes, and another infant died due to suspected cholangitis at the age of two months. According to previous reports, up to 80% of the children suffered from systemic hypertension [9, 10]. In our study, almost 50% of the pediatric patients showed systemic hypertension, and among two of them hypertension was the first manifestation. Thus, it is essential to consider ARPKD as a differential diagnosis while examining pediatric patients with hypertension.

Congenital hypothyroidism was detected in three patients (SC488, SC528, and SC574) via newborn mass screening. To the best of our knowledge, only one case of ARPKD with congenital hypothyroidism has been reported [16]. In Japan, nearly 100% of newborns undergo mass screening, and the incidence of congenital hypothyroidism is approximately 1/4000 [17]. Considering the incidence of both diseases, it is unlikely that these two diseases co-occurred incidentally. Elevation in thyroid-stimulating hormone levels was observed in all three patients; thus, the patients were diagnosed with primary congenital hypothyroidism rather than central congenital hypothyroidism. Primary congenital hypothyroidism is traditionally subdivided into thyroid dysgenesis and dyshormonogenesis [18]. Dyshormonogenesis was presumed to be the cause of hypothyroidism in three patients because normal ultrasonography findings of the thyroid were observed. ARPKD is a cilia-related disease, and polyductin/fibrocystin localizes in the primary cilia and basal bodies of the cell. Primary cilia have been found in the thyrocytes of humans [19], and a direct relationship between ciliogenesis and thyroid follicle activity has been revealed in the functional pathology of the thyroid gland [20]. RNA expression of the *PKHD1* gene in the thyroid gland was low but detectable; therefore, we assumed that the dysfunctions of primary cilia due to *PKHD1* gene mutation might lead to decreased follicular activity in the thyroid, which resulted in congenital hypothyroidism. More studies on primary cilia are needed to confirm the relationship between *PKHD1* and congenital hypothyroidism. Additionally, we need to carefully observe whether levothyroxine treatment of patients can be discontinued in the future.

In this study, we conducted a minigene assay to evaluate the splicing of *PKHD1*. Studies in large-scale cohorts revealed genotype–phenotype correlations for *PKHD1*; two truncating mutations display a severe phenotype associated with perinatal or neonatal death, and at least one missense mutation has been thought to be indispensable for survival during the neonatal period [9, 14]. However, the SC499 patient survived the neonatal period, and did not need renal replacement therapy until the age of six months, although she did not harbor a missense mutation. Only a few reports have described milder cases with no missense mutations [21, 22]. Both the nonsense and splice site variants in the minigene assay mainly expressed a transcript that skipped an exon with a multiple of three, resulting in in-frame mutations in both alleles. It has been suggested that nonsense-mediated mRNA decay (NMD) may play a role in defining the phenotype of patients with ARPKD [21]. It can be speculated that exon skipping in exons 25 and 41, which leads to in-frame mutations, may lead to the circumvention of NMD and contribute to neonatal survival. Additionally, an appropriate management during the neonatal period might have contributed to patient survival. Our results highlight the importance of functional analysis. Moreover, we call into question the fundamental belief that at least one missense mutation is necessary for survival through the perinatal or neonatal period.

On the other hand, three patients (SC293, SC324, and SC589) suffered from neonatal demise, and all of them had one missense mutation. One missense mutation does not guarantee perinatal and neonatal survival [14], and some studies have reported that missense mutations alter a splice enhancer motif that disrupts exon splicing, leading to aberrant *PKHD1* splicing [11]. We conducted a minigene assay for three cases to elucidate the mechanism involved in splicing that led to a severe phenotype. Nonsense mutations were detected in another allele of SC293 and SC589, and splice-site mutations were detected in SC324, which expressed a transcript that skipped exon 55 (88 bp), resulting in a truncating mutation. However, the minigene assay revealed that each missense mutation did not affect splicing. In SC324-1, a transcript with full-length exon 32 was not observed, but the splicing pattern was the same as that of the wild type; thus, we concluded that this mutation did not affect splicing. This is one of the limitations of minigene assays. Bergmann et al. demonstrated that the phenotypes due to *PKHD1* mutations cannot be explained on the basis of the genotype alone, but may also depend on the background of other genes, epigenetic factors, and environmental influences [9, 14]. Our minigene assay results support this idea. Although the lack of mutational hotspots and variety phenotypes in *PKHD1* hampers further analysis for genotype–phenotype correlations, more studies are needed to investigate the mechanism involved in severe phenotypes.

This study had some limitations. This analysis was based on cases referred to our institute for gene testing from hospitals in Japan, not a nationwide registry in Japan. Therefore, the number of included patients was relatively small, and we could not obtain patient information with a long follow-up period. Not all patients were able to undergo parental analysis for clinical reasons; therefore, some “uncertain significance” variants were included in this study (Supplementary Table 8). In addition, in vivo analyses, such as RNA sequencing, were not performed for the evaluation of alternative splicing because sufficient and high-quality RNA samples could not be obtained. As mentioned, in the minigene assay, a normal splicing pattern was not observed in wild-type SC324-1.

In conclusion, this is the first multicenter report of genetically diagnosed ARPKD in the Japanese population. We detected 20 novel mutations in *PKHD1*. Clinical manifestations ranged from cases that died in the neonatal period to those incidentally found in adulthood. The complication of congenital hypothyroidism might be associated with dyshormonogenesis in the thyroid due to *PKHD1* gene mutations. From the minigene assay, we propose the importance of functional analysis, and call into question the fundamental belief that at least one missense mutation is necessary for survival during the perinatal or neonatal period.

## Supplementary Information

Below is the link to the electronic supplementary material.

Supplementary file1 (DOCX 2749 kb)
