# Supplementary material for: Clinical features of autosomal recessive polycystic kidney disease in the Japanese population and analysis of splicing in PKHD1 gene for determination of phenotypes
Source: Clin Exp Nephrol. 2021 Sep 18;26(2):140–53. doi: 10.1007/s10157-021-02135-3 (PMC8770369; doi:10.1007/s10157-021-02135-3)
Supplement: Supplementary file 1 — Supplementary file1 (DOCX 2749 kb) [file 10157_2021_2135_MOESM1_ESM.docx]

Electronic supplementary material

Clinical features of autosomal recessive polycystic kidney disease (ARPKD) in the Japanese population and the analysis of splicing in *PKHD1* gene for determining the phenotypes

Contents

Supplementary Table 1. The gene list constructed using HaloPlex (version 2, 128 genes)

Supplementary Table 2. The gene list constructed using HaloPlex (version 4, 172 genes)

Supplementary Table 3. The gene list constructed using HaloPlex (version 5, 159 genes)

Supplementary Table 4. The gene list constructed using HaloPlex (version 6, 164 genes)

Supplementary Table 5. The gene list constructed using HaloPlex (version 7, 181 genes)

Supplementary Table 6. The gene list constructed using HaloPlex (version 8, 203 genes)

Supplementary Table 7. The gene list constructed using HaloPlex (version 9, 193 genes)

Supplementary Table 8. Pathogenicity predictions with the American College of

Medical Genetics guidelines

Supplementary Table 9. The *in-silico* evaluations for missense variants in the study

Supplementary Table 10. Primers used for cloning in minigene assay in each mutation

Supplementary Fig.1 Inserted site and the number of base pair into H492 vector with In-fusion cloning methods in minigene assay

Supplementary Fig.2 Inserted sequences into H492 vector with In- fusion cloning methods in minigene assay

Supplementary Fig.3 Results of direct sequencing for minigene transcript in each mutation

Supplementary Table 1. The gene list constructed using HaloPlex (version 2, 128 genes)

**
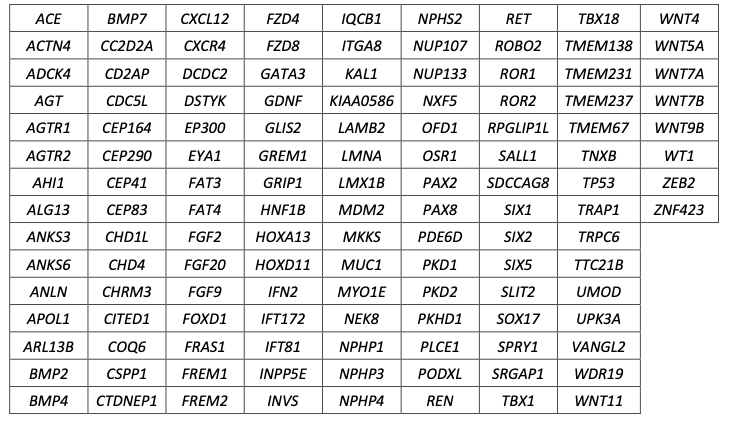
**

Supplementary Table 2. The gene list constructed using HaloPlex (version 4, 172genes)

**
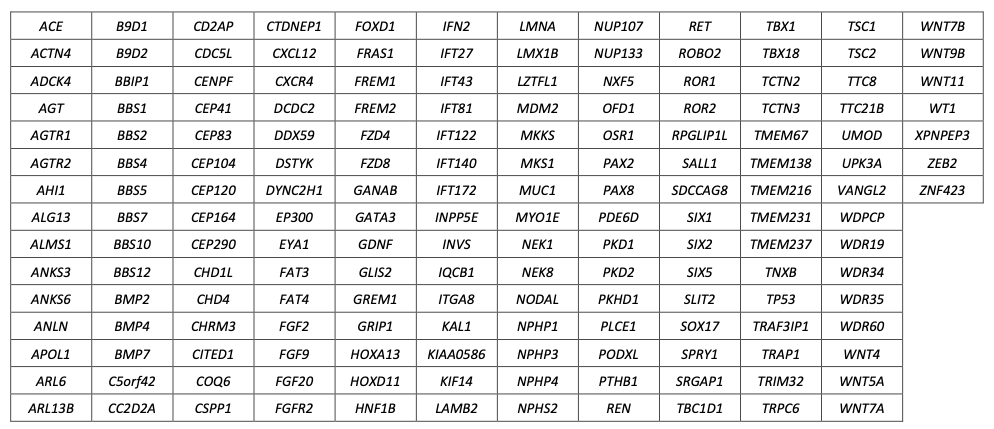
**

Supplementary Table 3. The gene list constructed using HaloPlex (version 5, 159 genes)

| *ACE* | *BBS1* | *CEP41* | *DYNC2H1* | *GANAB* | *IFT80* | *LMNA* | *PDE6D* | *SIX1* | *TMEM107* | *WDPCP* |
| --- | --- | --- | --- | --- | --- | --- | --- | --- | --- | --- |
| *AGT* | *BBS2* | *CEP83* | *DZIP1L* | *GATA3* | *IFT81* | *LMX1B* | *PIBF1* | *SIX2* | *TMEM138* | *WDR19* |
| *AGTR1* | *BBS4* | *CEP104* | *EP300* | *GDNF* | *IFT122* | *LZTFL1* | *PKD1* | *SIX5* | *TMEM216* | *WDR34* |
| *AGTR2* | *BBS5* | *CEP120* | *EVC* | *GLIS2* | *IFT140* | *MKKS* | *PKD2* | *SLIT2* | *TMEM231* | *WDR35* |
| *AHI1* | *BBS7* | *CEP164* | *EVC2* | *GLIS3* | *IFT172* | *MKS1* | *PKHD1* | *SOX17* | *TMEM237* | *WDR60* |
| *ALG9* | *BBS10* | *CEP290* | *EXOC4* | *GRIP1* | *INPP5E* | *MUC1* | *PTHB1* | *SPRY1* | *TNXB* | *WNT4* |
| *ALMS1* | *BBS12* | *CHD1L* | *EXOC8* | *GRLF1* | *INVS* | *NEK1* | *REN* | *SRGAP1* | *TRAF3IP1* | *WT1* |
| *ANKS3* | *C2CD3* | *CHD4* | *EYA1* | *HNF1B* | *IQCB1* | *NEK8* | *RET* | *TBC1D1* | *TRIM32* | *XPNPEP3* |
| *ANKS6* | *C5orf42* | *CHRM3* | *FAN1* | *HOXA13* | *ITGA8* | *NPHP1* | *ROBO2* | *TBC1D32* | *TSC1* | *ZNF423* |
| *ARL6* | *C21orf2* | *CITED1* | *FGF9* | *HPRT1* | *JAG1* | *NPHP3* | *RPGLIP1L* | *TBX1* | *TSC2* |  |
| *ARL13B* | *CC2D2A* | *CSPP1* | *FGF20* | *HYLS1* | *KAL1* | *NPHP4* | *SALL1* | *TBX18* | *TTBK2* |  |
| *ATXN10* | *CCDC28B* | *CTDNEP1* | *FGFR2* | *ICK* | *KIAA0586* | *OFD1* | *SARS2* | *TCTN1* | *TTC8* |  |
| *B9D1* | *CDC5L* | *DCDC2* | *FRAS1* | *IFN2* | *KIF7* | *PAX2* | *SCLT1* | *TCTN2* | *TTC21B* |  |
| *B9D2* | *CENPF* | *DDX59* | *FREM1* | *IFT27* | *KIF14* | *PAX8* | *SDCCAG8* | *TCTN3* | *UMOD* |  |
| *BBIP1* | *CEP19* | *DSTYK* | *FREM2* | *IFT43* | *LIFR* | *PBX1* | *SEC61A1* | *TMEM67* | *VANGL2* |  |

Supplementary Table 4. The gene list constructed using HaloPlex (version 6, 164genes)

**
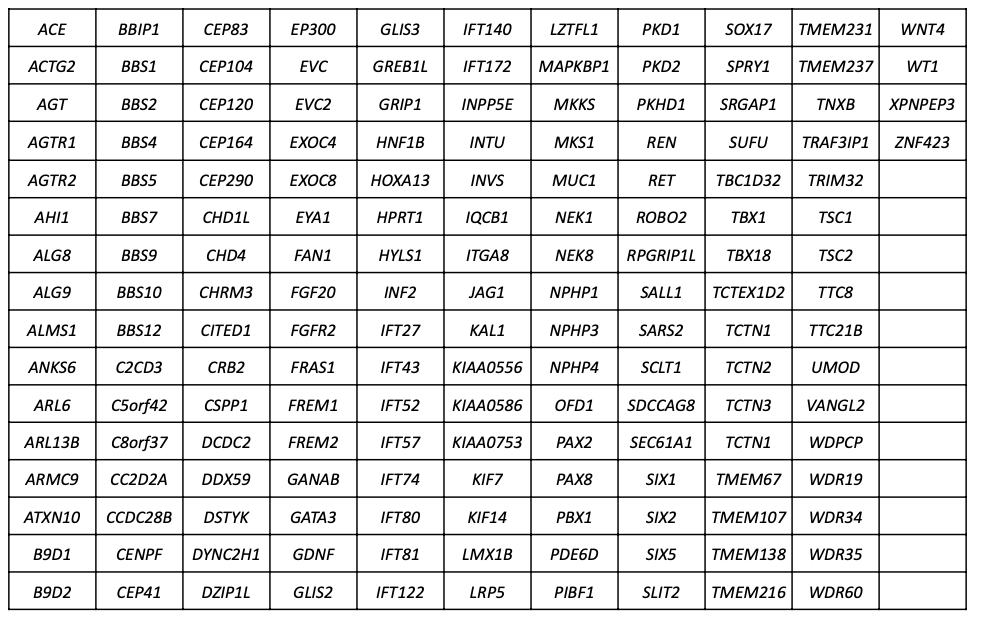
**

Supplementary Table 5. The gene list constructed using HaloPlex (version 7, 181 genes)

| *ACE* | *BBS1* | *CEP83* | *EP300* | *GFRA1* | *IFT80* | *LRIG2* | *PIBF1* | *SOX11* | *TNXB* | *ZNF423* |
| --- | --- | --- | --- | --- | --- | --- | --- | --- | --- | --- |
| *ACTG2* | *BBS2* | *CEP104* | *EVC* | *GLIS2* | *IFT81* | *LRP5* | *PKD1* | *SOX17* | *TRAF3IP1* |  |
| *AGT* | *BBS4* | *CEP120* | *EVC2* | *GLIS3* | *IFT122* | *LZTFL1* | *PKD2* | *SPRY1* | *TRIM32* |  |
| *AGTR1* | *BBS5* | *CEP164* | *EXOC4* | *GPC3* | *IFT140* | *MAPKBP1* | *PKHD1* | *SRGAP1* | *TSC1* |  |
| *AGTR2* | *BBS7* | *CEP290* | *EXOC8* | *GREB1L* | *IFT172* | *MKKS* | *REN* | *SUFU* | *TSC2* |  |
| *AHI1* | *BBS9* | *CHD1L* | *EYA1* | *GREM1* | *INPP5E* | *MKS1* | *RET* | *TBC1D32* | *TTC8* |  |
| *ALG8* | *BBS10* | *CHD4* | *FAN1* | *GRIP1* | *INTU* | *MUC1* | *ROBO2* | *TBX1* | *TTC21B* |  |
| *ALG9* | *BBS12* | *CHD7* | *FGF20* | *HNF1B* | *INVS* | *NEK1* | *RPGRIP1L* | *TBX18* | *UMOD* |  |
| *ALMS1* | *BICC1* | *CHRM3* | *FGFR1* | *HOXA13* | *IQCB1* | *NEK8* | *SALL1* | *TCTEX1D2* | *UPK3A* |  |
| *ANKS6* | *C2CD3* | *CITED1* | *FGFR2* | *HPRT1* | *ITGA8* | *NOTCH2* | *SARS2* | *TCTN1* | *VANGL2* |  |
| *ARL3* | *C5orf42* | *CRB2* | *FRAS1* | *HPSE2* | *JAG1* | *NPHP1* | *SCLT1* | *TCTN2* | *WDPCP* |  |
| *ARL6* | *C8orf37* | *CSPP1* | *FREM1* | *HYLS1* | *KAL1* | *NPHP3* | *SDCCAG8* | *TCTN3* | *WDR19* |  |
| *ARL13B* | *CC2D2A* | *DCDC2* | *FREM2* | *INF2* | *KIAA0556* | *NPHP4* | *SEC61A1* | *TMEM67* | *WDR34* |  |
| *ARMC9* | *CCDC28B* | *DDX59* | *GANAB* | *IFT27* | *KIAA0586* | *OFD1* | *SIX1* | *TMEM107* | *WDR35* |  |
| *ATXN10* | *CDC5L* | *DNAJB11* | *GATA3* | *IFT43* | *KIAA0753* | *PAX2* | *SIX2* | *TMEM138* | *WDR60* |  |
| *B9D1* | *CDKN1C* | *DSTYK* | *GDF11* | *IFT52* | *KIF7* | *PAX8* | *SIX5* | *TMEM216* | *WNT4* |  |
| *B9D2* | *CENPF* | *DYNC2H1* | *GDNF* | *IFT57* | *KIF14* | *PBX1* | *SLIT2* | *TMEM231* | *WT1* |  |
| *BBIP1* | *CEP41* | *DZIP1L* | *GEN1* | *IFT74* | *LMX1B* | *PDE6D* | *SOX9* | *TMEM237* | *XPNPEP3* |  |

Supplementary Table 6. The gene list constructed using HaloPlex (version 8, 203 genes)


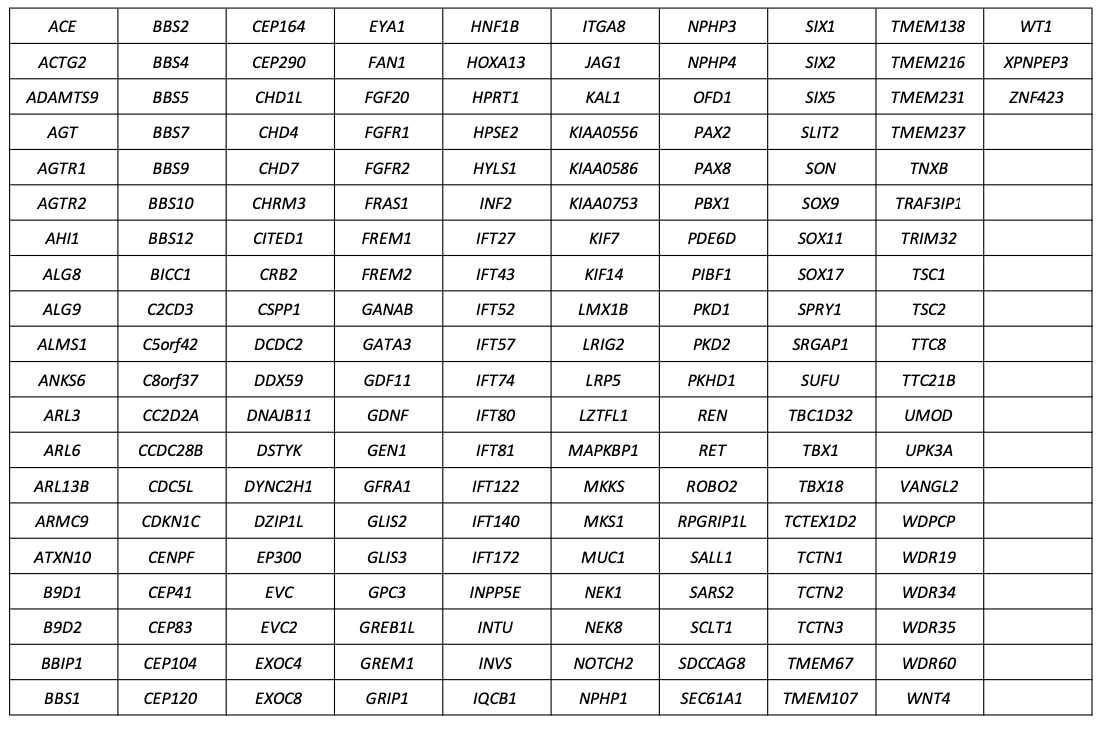


Supplementary Table 7. The gene list constructed using HaloPlex (version 9, 193 genes)

| *ACE* | *BBS2* | *CEP164* | *EVC2* | *GPC3* | *IFT172* | *MKS1* | *RET* | *TBC1D32* | *TTC21B* |
| --- | --- | --- | --- | --- | --- | --- | --- | --- | --- |
| *ACTG2* | *BBS4* | *CEP290* | *EXOC4* | *GREB1L* | *INPP5E* | *MUC1* | *ROBO2* | *TBX1* | *UMOD* |
| *ADAMTS9* | *BBS5* | *CHD1L* | *EXOC8* | *GREM1* | *INTU* | *NEK1* | *RPGRIP1L* | *TBX6* | *UPK3A* |
| *AGT* | *BBS7* | *CHD4* | *EYA1* | *GRIP1* | *INVS* | *NEK8* | *SALL1* | *TBX18* | *VANGL2* |
| *AGTR1* | *BBS9* | *CHD7* | *FAM149B1* | *HNF1B* | *IQCB1* | *NOTCH2* | *SARS2* | *TCTEX1D2* | *WDPCP* |
| *AGTR2* | *BBS10* | *CHRM3* | *FAN1* | *HOXA11* | *ITGA8* | *NPHP1* | *SCLT1* | *TCTN1* | *WDR19* |
| *AHI1* | *BBS12* | *CHRNA3* | *FGF20* | *HOXA13* | *JAG1* | *NPHP3* | *SDCCAG8* | *TCTN2* | *WDR34* |
| *ALG8* | *BICC1* | *CLCN5* | *FGFR1* | *HPRT1* | *KAL1* | *NPHP4* | *SEC61A1* | *TCTN3* | *WDR35* |
| *ALG9* | *C2CD3* | *CITED1* | *FGFR2* | *HPSE2* | *KIAA0556* | *NRIP1* | *SIX1* | *TMEM67* | *WDR60* |
| *ALMS1* | *C5orf42* | *CRB2* | *FRAS1* | *HYLS1* | *KIAA0586* | *OCRL* | *SIX2* | *TMEM107* | *WNT4* |
| *ANKS6* | *C8orf37* | *CSPP1* | *FREM1* | *INF2* | *KIAA0753* | *OFD1* | *SIX5* | *TMEM138* | *WT1* |
| *ARL3* | *CC2D2A* | *DCDC2* | *FREM2* | *IFT27* | *KIF7* | *PAX2* | *SLC20A1* | *TMEM216* | *XPNPEP3* |
| *ARL6* | *CCDC28B* | *DDX59* | *GANAB* | *IFT43* | *KIF14* | *PAX8* | *SLIT2* | *TMEM231* | *ZNF423* |
| *ARL13B* | *CDC5L* | *DNAJB11* | *GATA3* | *IFT52* | *LMX1B* | *PBX1* | *SON* | *TMEM237* |  |
| *ARMC9* | *CDKN1C* | *DSTYK* | *GDF11* | *IFT57* | *LRIG2* | *PDE6D* | *SOX9* | *TNXB* |  |
| *ATXN10* | *CENPF* | *DYNC2H1* | *GDNF* | *IFT74* | *LRP5* | *PIBF1* | *SOX11* | *TRAF3IP1* |  |
| *B9D1* | *CEP41* | *DYNC2LI1* | *GEN1* | *IFT80* | *LZTFL1* | *PKD1* | *SOX17* | *TRIM32* |  |
| *B9D2* | *CEP83* | *DZIP1L* | *GFRA1* | *IFT81* | *MAFB* | *PKD2* | *SPRY1* | *TSC1* |  |
| *BBIP1* | *CEP104* | *EP300* | *GLIS2* | *IFT122* | *MAPKBP1* | *PKHD1* | *SRGAP1* | *TSC2* |  |
| *BBS1* | *CEP120* | *EVC* | *GLIS3* | *IFT140* | *MKKS* | *REN* | *SUFU* | *TTC8* |  |

Supplementary Table 8.

Pathogenicity predictions with the American College of Medical Genetics guidelines

| Family | Patient |  | ACMG | | |
| --- | --- | --- | --- | --- | --- |
|  |  |  | Category | Evidence of pathogenicity | Classification |
| 1 | SC272 | c.2507T>C | PS1 PM3 PP3, PP4 | 1 Strong 1 Moderate 2 Supporting | Likely pathogenic |
|  |  | c.9008C>T | PS1 PM2, PM3 PP3, PP4 | 1 Strong 2 Moderate 1 Supporting | Pathogenic |
| 2 | SC282 | c.11G>A | PVS1 PM2 PP3, PP4 | Very strong 1 Moderate 2 Supporting | Pathogenic |
|  |  | c.2507T>C | PS1  PM3 PP3, PP4 | 1 Strong  1 Moderate 2 Supporting | Likely pathogenic |
| 3 | SC293 | c.7113T>G | PVS1 PM2, PM4 PP3, PP4 | Very strong 2 Moderate 2 supporting | Pathogenic |
|  |  | c.9533G>T | PM2, PM5 PP3, PP4 | 2 Moderate 2 Supporting | Likely pathogenic |
| 4 | SC324 | c.3944T>G | PM2, PM3 PP3, PP4 | 2 Moderate 2 Supporting | Likely pathogenic |
|  |  | c.8555-2A>C | PM2, PM3 PP3, PP4 | 2 Moderate 2 Supporting | Likely pathogenic |
| 5 | SC331 | c.7396G>T | PVS1 PM2 PP3, PP4 | Very strong 1 Moderate 2 supporting | Pathogenic |
|  |  | c.8859G>C | PM2  PP3, PP4 | 1 Moderate 2 Supporting | Uncertain significance |
| 6 | SC365 | c.274C>T | PS1 PM2 PP4 | 1 Strong 1 Moderate 1 Supporting | Likely pathogenic |
|  |  | c.9319C>T | PVS1 PS1 PM2 PP4, PP5 | Very strong 1 Strong 1 Moderate 1 Supporting | Pathogenic |
| 7 | SC410 | c.3467C>T | PS1 PM1, PM2 PP4  BP4 | 1 Strong 2 Moderate 1 Supporting  1 Supporting (Benign) | Likely pathogenic |
|  |  | c.5585C>A | PVS1 PM2, PM3, PM4 PP3, PP4 | Very strong 3 Moderate 2 Supporting | Pathogenic |
| 8 | SC432 | c.1486C>T | PVS1 PS1 PM3,  PP3, PP4, PP5  BS1 | Very strong 1 Strong 1 Moderate 3 Supporting  1 Strong (benign) | Pathogenic |
|  |  | c.6840G>A | PVS1 PM2, PM3 PP3, PP4 | Very strong 2 Moderate 2 Supporting | Pathogenic |
| 9 | SC443 | c.2507T>C | PS1 PM2, PM3 PP3, PP4 | 1 Strong 2 Moderate 2 Supporting | Pathogenic |
|  |  | c.8566A>T | PVS1 PM2, PM3 PP3, PP4 | Very strong 2 Moderate 2 Supporting | Pathogenic |
| 10 | SC481 | c.11611T>C | PS1 PM2 PP1, PP3, PP4 | 1 Strong 1 Moderate 3 Supporting | Likely pathogenic |
|  |  | c.11881C>T | PVS1 PS1 PM2 PP1, PP4 | Very strong 1 Strong 1 Moderate 2 Supporting | Pathogenic |
|  | Brother | c.11611T>C | PS1 PM2 PP1, PP3, PP4 | 1 Strong 1 Moderate 3 Supporting | Likely pathogenic |
|  |  | c.11881C>T | PVS1 PS1 PM2 PP1, PP4 | Very strong 1 Strong 1 Moderate 2 Supporting | Pathogenic |
| 11 | SC488 | c.977-3C>G | PM2 PP4 | 1 Moderate 1 Supporting | Uncertain significance |
|  |  | c.10180T>C | PS1 PM2 PP3, PP4 | 1 Strong 1 Moderate 2 Supporting | Likely pathogenic |
| 12 | SC494 | c.5174G>C | PM2 PP3, PP4 | 1 Moderate 2 Supporting | Uncertain significance |
|  |  | (PKHD1 exons 54-55) x1 | PVS1 PM2 PP4 | Very strong 1 Moderate 1 Supporting | Likely pathogenic |
| 13 | SC498 | c.5174G>C | PM2 PP3, PP4 | 1 Moderate 2 Supporting | Uncertain significance |
|  |  | c.7867delT | PVS1 PM2 PP4 | Very strong 1 Moderate 1 Supporting | Pathogenic |
| 14 | SC499 | c.2713C>T | PVS1 PS1 PM2, PM3 PP3, PP4 | Very strong 1 Strong 2 Moderate 2 Supporting | Pathogenic |
|  |  | c.6801+G>A | PM2, PM3 PP4 | 2 Moderate 1 Supporting | Uncertain significance |
| 15 | SC528 | c.7237C>T | PM1, PM2, PM3 PP4 | 3 Moderate 1 Supporting | Likely pathogenic |
|  |  | c.8893T>C | PS1 PM2, PM3 PP3, PP4 | 1 Strong 1 Moderate 2 Supporting | Pathogenic |
| 16 | SC529 | c.1836+1G>A | PM2, PM3 PP4 | Very strong | Likely pathogenic |
|  |  | c.5935G>A | PS1 PM2, PM3 PP3, PP4 | 1 Strong 2 Moderate 2 Supporting | Pathogenic |
| 17 | SC567 | c.5174G>C | PM2, PM3 PP3, PP4 | 2 Moderate 2 Supporting | Likely pathogenic |
|  |  | c.11456delT | PVS1 PM2, PM4 PP4 | Very strong 2 Moderate 1 supporting | Pathogenic |
| 18 | SC574 | c.4292G>A | PS1 PM1, PM2 PP3, PP4, PP5 | 1 Strong 2 Moderate 3 Supporting | Pathogenic |
|  |  | c.9533G>A | PM1, PM2 PP3, PP4 | 2 Moderate 2 Supporting | Likely pathogenic |
| 19 | SC583 | c.865C>T | PVS1 PM2, PM3 PP3, PP4 | Very strong 2 Moderate 2 Supporting | Pathogenic |
|  |  | c.5935G>A | PS1 PM2, PM3 PP3, PP4 | 1 Strong 2 Moderate 2 Supporting | Pathogenic |
| 20 | SC589 | c.983G>A | PS1 PM1, PM2, PM3 PP3, PP4 | 1 Strong 3 Moderate 2 Supporting | Pathogenic |
|  |  | c.8011C>T | PVS1 PS1 PM2, PM3 PP3, PP4, PP5 | Very strong 1 Strong 2 Moderate 3 Supporting | Pathogenic |
| 21 | SC601 | c.1241A>C | PM2 PP3, PP4 | 1 Moderate 1 Supporting | Uncertain significance |
|  |  | c.5174G>C | PM2, PM3 PP3, PP4 | 2 Moderate 2 Supporting | Likely pathogenic |
| 22 | SC619 | c.2725C>T | PVS1 PS1 PM2, PM3 PP4, PP5 | Very strong 1 Strong 2 Moderate 2 Supporting | Pathogenic |
|  |  | c.5935G>A | PS1 PM2, PM3 PP3, PP4 | 1 Strong 2 Moderate 2 Supporting | Pathogenic |
| 23 | SC637 | c.11G>A | PVS1  PS1 PM2, PM3 PP3, PP4, PP5 | Very strong  1 strong 2 Moderate 3 supporting | Pathogenic |
|  |  | c.6794A>T | PM1, PM2, PM3  PP3, PP4 | 2 Moderate  1 Supporting | Likely Pathogenic |
| 24 | SC681 | c.2507T>C | PS1  PM3 PP3, PP4 | 1 Strong  1 Moderate 2 Supporting | Likely pathogenic |
|  |  | c.10414T>G | PM2, PM3  PP3, PP4 | 2 Moderate 2 Supporting | Likely Pathogenic |
| 25 | SC697 | c.7867delT | PVS1 PM2 PP4 | Very strong 1 Moderate 1 Supporting | Pathogenic |
|  |  | (PKHD1 exon 50) x1 | PVS1 PM2 PP4 | Very strong 1 Moderate 1 Supporting | Pathogenic |
| 26 | SC704 | c.5935G>A | PS1 PM2, PM3 PP3, PP4 | 1 Strong 2 Moderate 2 Supporting | Pathogenic |
|  |  | c.7867delT | PVS1 PM2, PM3 PP4 | Very strong 2 Moderate 1 Supporting | Pathogenic |
| 27 | SC746 | c.9746G>C | PM2, PM3  PP4 | 2 Moderate  1 Supporting | Uncertain significance |
| 28 | SC756 | c.9107T>G | PM1, PM2, PM3  PP4 | 3 Moderate  1 Supporting | Likely pathogenic |
| 29 | SC772 | c.1396G>A | PM2, PM3  PP3, PP4 | 2 Moderate  2 Supporting | Likely pathogenic |
|  |  | c.6794A>T | PM1, PM2, PM3  PP3, PP4 | 3 Moderate  2 Supporting | Likely pathogenic |
| 30 | SC791 | c.2507T>C | PS1  PM3 PP3, PP4 | 1 Strong  1 Moderate 2 Supporting | Likely pathogenic |
|  |  | c.5780G>A | PM2  PP4 | 1 Moderate  1 Supporting | Uncertain significance |
| 31 | SC793 | c.1690C>T | PVS1  PS1  PM2, PM3  PP3, PP4 | Very strong  1 Strong  2 Moderate  2 Supporting | Pathogenic |
|  |  | c.2507T>C | PS1  PM3 PP3, PP4 | 1 Strong  1 Moderate 2 Supporting | Likely pathogenic |

Supplementary Table 9. The in-silico evaluations for missense variants in the study

| Genotype | Exon | Amino acid | CADD | PROVEAN | SIFT | Polyphen2 | Mutation Taster | Patient |
| --- | --- | --- | --- | --- | --- | --- | --- | --- |
| c.274C>T | 4 | p.Arg92Trp | 31 | N | D | P | N | SC365 |
| c.983G>A | 14 | p.Arg328Gln | 34 | D | D | D | P | SC589 |
| c.1396G>A | 16 | p.Gly466Arg | 25.9 | D | D | D | D | SC772 |
| c.1421A>C | 16 | p.His474Pro | 26.2 | D | D | D | D | SC601 |
| c.2507T>C | 24 | p.Val836Ala | 22.1 | D | D | P | N | SC272, SC282, SC443, SC681, SC791, SC793 |
| c.3467C>T | 30 | p.Ser1156Leu | 16.39 | N | D | B | N | SC410 |
| c.3944T>G | 32 | p.Leu1315Arg | 24 | D | D | D | N | SC324 |
| c.4292G>A | 32 | p.Cys1431Tyr | 24.3 | D | D | P | P | SC574 |
| c.5174G>C | 32 | p.Trp1725Ser | 23.8 | D | T | P | D | SC494. SC498. SC567, SC601 |
| c.5780G>A | 36 | p.Arg1927Lys | 24 | N | T | P | N | SC791 |
| c.5935G>A | 37 | p.Gly1979Arg | 34 | D | D | D | D | SC529, SC583, SC619, SC704 |
| c.6794A>T | 41 | p.His2265Leu | 24.9 | D | D | D | D | SC637, SC772 |
| c.7237C>T | 46 | p.Arg2413Cys | 26.1 | D | D | P | D | SC528 |
| c.8859G>C | 57 | p.Leu2953Phe | 26.5 | D | D | D | D | SC331 |
| c.8893T>C | 57 | p.Cys2965Arg | 22.5 | D | D | D | D | SC528 |
| c.9008C>T | 58 | p.Ser3003Phe | 24.9 | N | T | P | D | SC272 |
| c.9107T>G | 58 | p.Val3036Gly | 23 | D | D | P | N | SC756 |
| c.9533G>T | 58 | p.Gly3178Val | 26.3 | D | D | D | D | SC293, SC574 |
| c.9764G>C | 58 | p.Trp3255Ser | 24.5 | D | T | P | D | SC746 |
| c.10180T>C | 61 | p.Cys3394Arg | 26.3 | D | D | D | D | SC488 |
| c.10414T>G | 61 | p.Cys3472Gly | 22.8 | D | D | D | D | SC681 |
| c.11611T>C | 65 | p.Trp3871Arg | 25.4 | D | D | P | N | SC481 |

Supplementary Table 10. Primers used for cloning in minigene assay in each mutation

| SC499-1 Forward | CCGTGCTTTGTTAGCCCTCTTTTGGCAATCCACTTG |
| --- | --- |
| SC499-1 Reverse | TCGATGTTAACGCTACCACCAGGTAGAAGCATAAATCA |
| SC499-2 Forward | CCGTGCTTTGTTAGCCCAGTTGGAAATCTTGACAGC |
| SC499-2 Reverse | TCGATGTTAACGCTACTTGGCCAACATCCAAGTTTG |
| SC293-2 Forward | CCGTGCTTTGTTAGCATCCCCAAAGTTTGCCTCTTTC |
| SC293-2 Reverse | TCGATGTTAACGCTACTTGAGGCCAGGAGTTTGAG |
| SC324-1 Forward | CCGTGCTTTGTTAGCAAGTGGTGGCTTCTGGGATA |
| SC324-1 Reverse | TCGATGTTAACGCTAGGTGAAGGCATTTACGTTCTTA |
| SC324-2 Forward | CCGTGCTTTGTTAGCGAGTGGTCTATTTTCCCACCTTTTA |
| SC324-2 Reverse | TCGATGTTAACGCTATGAGGTACCTTTTCAGCTTAGACA |
| SC589-1 Forward | CCGTGCTTTGTTAGCGCCTCAGCTTCTGTTTGCTC |
| SC589-1 Reverse | TCGATGTTAACGCTAAGGCTTTGCTCCCTCATGTA |
